# High Incidence of Mammalian Orthoreovirus Identified by Environmental Surveillance in Taiwan

**DOI:** 10.1371/journal.pone.0142745

**Published:** 2015-11-10

**Authors:** Matthew C. Y. Lim, Ya-Fang Wang, Sheng-Wen Huang, Jyh-Yuan Yang, Jen-Ren Wang

**Affiliations:** 1 Department of Medical Laboratory Science and Biotechnology, College of Medicine, National Cheng Kung University, Tainan, Taiwan; 2 Center of Infectious Disease and Signaling Research, National Cheng Kung University, Tainan, Taiwan; 3 Department of Pathology, National Cheng Kung University Hospital, Tainan, Taiwan; 4 Center for Research, Diagnostics and Vaccine Development, Centers for Disease Control, Ministry of Health and Welfare, Taipei, Taiwan; 5 National Institute of Infectious Diseases and Vaccinology, National Health Research Institutes, Tainan, Taiwan; The University of Hong Kong, HONG KONG

## Abstract

Wild poliovirus (WPV) persists in diverse locales worldwide, spreading outward from endemic areas. In response to the international threat of WPV transmission and changes in the national vaccination policy, we established an environmental surveillance system to monitor the circulation of wild and vaccine-related poliovirus in Taiwan. From July 2012 to December 2013, we collected sewage specimens every month from 10 sewage treatment plants located throughout Taiwan. The specimens were concentrated by the two-phase separation method and then inoculated into L20B, RD, and A549 cells for virus isolation. Viral isolates were identified and serotyped by immunofluorescence assay or molecular analysis. A total of 300 sewage samples were collected, and the results showed 163 samples (54.3%) were positive for virus, and 268 isolates were identified. Among these, 75 samples (25%) were positive for enterovirus (EV), but no poliovirus was found. In addition, 92 isolates were identified as enteroviruses and the most common serotypes were coxsackievirus B4, coxsackievirus B3, and coxsackievirus B2. Interestingly, 102 (34%) and 82 (27.3%) specimens were positive for mammalian orthoreovirus (MRV) and adenovirus, respectively. This study confirmed that sewage surveillance can be a useful additional modality for monitoring the possible presence of wild-type or vaccine-derived poliovirus in wastewater, and can indicate the current types of viruses circulating in the population. Furthermore, since MRV was found in children with acute necrotizing encephalopathy and meningitis, the high incidence of MRV detected by environmental surveillance warrants further investigation.

## Introduction

The World Health Organization (WHO) Global Polio Eradication Initiative (GPEI) was established in 1988 and successfully prevented wild-type poliovirus (WPV) transmission in the Americas, the Western Pacific (WPR), and Europe (EUR) [1–3]. The Southeast Asia Region (SEAR), home to a quarter of the world's population, was also certified polio-free in March 2014 [4]. WHO certified Taiwan, along with the entire WPR, as polio-free in 2000 and Taiwan changed its immunization strategy from oral (OPV) to inactivated polio vaccine (IPV) in 2010. To date, WPV remains endemic in Afghanistan, Nigeria, and Pakistan. Numerous outbreaks in heretofore polio-free regions have been reported recently in China (2011), Somalia (2013), Ethiopia (2013), and Kenya (2013) caused by importation [5–7]. Besides WPV, cases of circulating vaccine-derived poliovirus (cVDPV) causing acute flaccid paralysis (AFP) have risen since 2000, and have been identified in eight countries in 2013 and in two countries in May 2014 [8].

Normally, acute flaccid paralysis (AFP) surveillance is the gold standard for poliovirus surveillance in eradication initiatives; under certain circumstances, environment surveillance is also employed to monitor the circulation of poliovirus in populations in order to better understand its evolution and transmission [9–13]. For instance, although certified as polio-free in 2002, Israel isolated WPV in routine environmental sewage samples in early February 2013, and immediate steps were taken to implement national supplementary immunization with OPV to prevent its spread [14]. Recently, the WHO included environmental poliovirus surveillance in a new strategic plan as part of its global eradication initiative to supplement AFP surveillance [15]. In Taiwan, AFP surveillance has long been established for poliovirus surveillance of the population, but environmental surveillance is not routinely performed.

Besides poliovirus in populations, enteroviruses, adenoviruses, reoviruses, and noroviruses are often found in environmental raw sewage [16–19]. These groups of viruses can cause a broad range of asymptomatic to severe gasterointestinal or respiratory infections [20], or even more acute conditions such as meningitis and paralysis [21], thus constituting a considerable public health problem in the community. Among these fecal-oral viral pathogens, reovirus is usually the most abundant virus detected in environmental water [22, 23]. Mammalian orthoreovirus (MRV), which belongs to the family *Reoviridae* and the genus *Orthoreovirus*, are a common class of enteric viruses capable of infecting a broad range of mammalian species, including humans. Previous studies have indicated that reoviruses have a high endemic infection rate in humans [24] and seroconversion was found in more than 70% of 4-year-old children [25]. Although reovirus infection in humans usually induces mild respiratory or gastrointestinal symptoms, there are reports of human reovirus-associated neurological disease [26, 27]. Several studies also described the isolation of reovirus strains directly from cerebrospinal fluid (CSF) or neural tissues obtained from patients with meningitis or encephalitis [28–31]. In addition, immunocompromised, pediatric, and elderly populations may become susceptible to severe bacterial respiratory disease due to an initial reovirus infection [32, 33].

In response to the international threat of WPV importation and the changes to the national vaccination policy, we adopted the WHO guidelines for environmental surveillance of circulation in Taiwan. Two-phase Dextran 40/Polyethylene glycol (PEG) separation and cell culture were performed to monitor environmental viral circulation [15]. We successfully isolated enteroviruses, adenoviruses, and mammalian orthoreoviruses, but no poliovirus was detected in sewage collected islandwide. Our results showed a high incidence of MRV, which may cause human disease, and thus further research is warranted.

## Materials and Methods

### Collection of sewage from treatment plant

**Fig 1:**
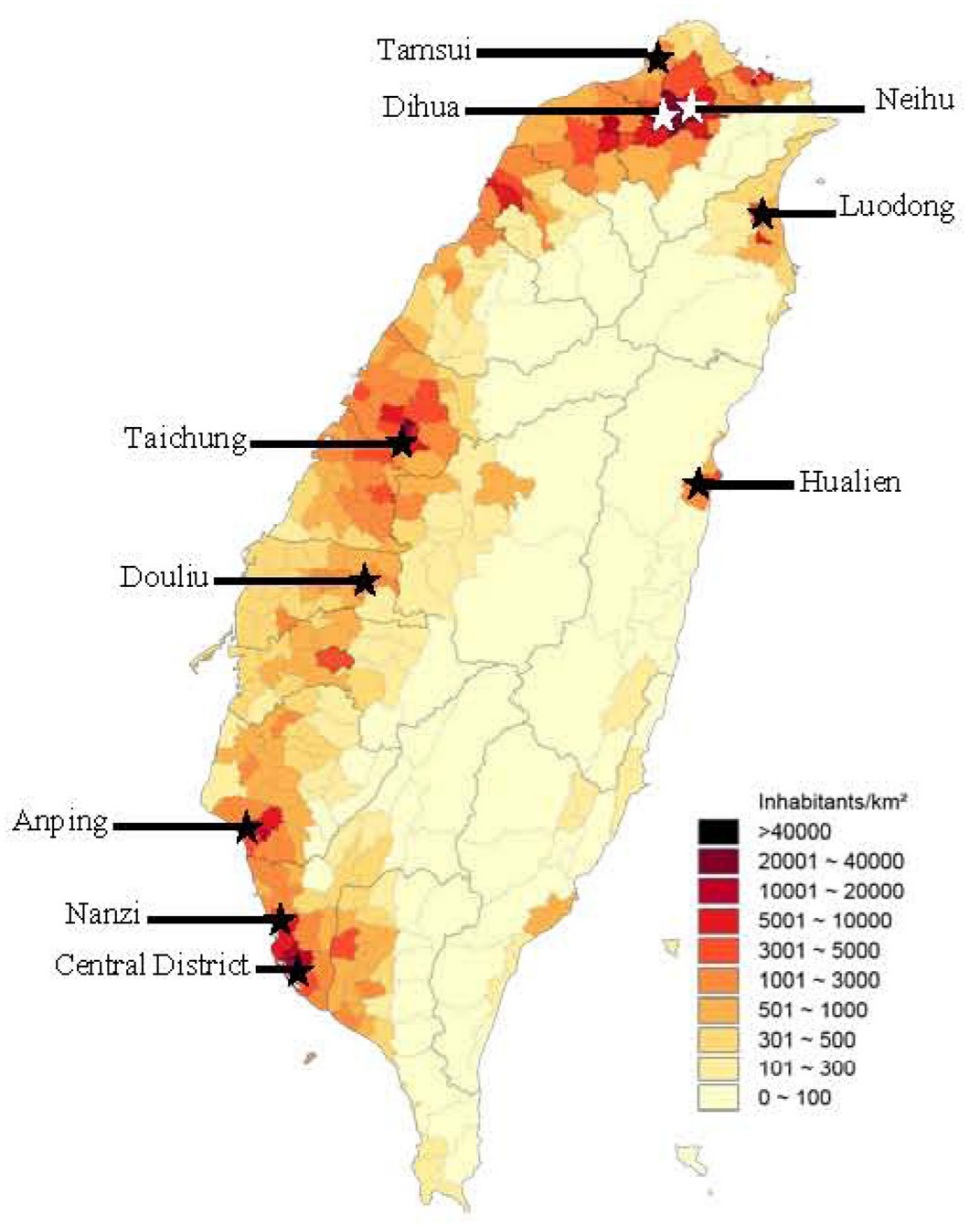
Location of sewage collection sites and county population densities in Taiwan from July 2012 to December 2013. (★) indicates the sewage treatment plants studied. Modified from http://commons.wikimedia.org/wiki/File%3APopulation_density_of_Taiwan_by_district.svg under a Creative Commons Attribution Share-Alike 3.0 (CC-BY-SA 3.0) license, with permission from Wikimedia Commons, 2013.

Specimens were collected at monthly intervals from July to December in 2012 and two separate samples in different weeks each month were collected in 2013 from ten representative wastewater treatment plants located in northern, central, southern, and eastern Taiwan (Fig 1). One liter of specimen from the raw sewage inlet of a treatment plant was transported to the study site at National Cheng Kung University. Specimens were kept at 4°C in transit and before inoculation into cell culture.

### Concentration of sewage specimens

Two-phase separation was conducted according to the World Health Organization (WHO) guidelines for environmental surveillance of poliovirus circulation and the *WHO Polio Laboratory Manual* as follows. A volume of 500 milliliters of specimen was centrifuged for 10 min with 1000g at 4°C, and pellets were kept at 4°C. The supernatant was transferred to a new bottle and the pH was adjusted to neutral (7.0–7.5). Next, 39.5 ml of 22% dextran, 287 ml of 29% PEG6000, and 35 ml of 5N NaCl were added to the supernatant and shaken for 1 hour. The supernatant was transferred to a sterile conical separation funnel and allowed to stand overnight. The entire lower layer and interphase were harvested and mixed with pellets, followed by addition of 20% volume of chloroform and centrifugation for extraction. The upper water phase was collected and penicillin/streptomycin was added. Then 200 μl of concentrated specimen was inoculated into cell lines immediately or frozen at -80°C for later use.

### Inoculation of sewage specimens

Human rhabdomyosarcoma (RD) cells (ATCC CCL-136^™^), recombinant murine cells L20B (mouse fibroblast cells that express the human poliovirus receptor) (obtained from Dr. Bruce Thorley, WHO polio reference laboratory in Australia) and human lung adenocarcinoma epithelial (A549) cells (ATCC CCL-185^™^) (added for viral isolation in 2013) were used for isolation of environmental viruses [34]. Concentrated specimens (200 μl) were inoculated into each cell culture tube, incubated at 35°C, and examined for cytopathic effects (CPE) daily. At day 7, negative CPE tubes were blind passed to a new cell culture tube and observed daily until day 14.

### Typing by immunofluorescent stain (IFA)

Positive CPE specimens were stained by Pan-Enterovirus (PanEV) Blend antibody and respiratory virus screen antibody (LIGHT DIAGNOSTICS^™^, Millipore) (only for positive specimens in A549 cells) for enterovirus or respiratory virus identification. The pan-enterovirus positive specimens were subtyped by enterovirus screening: echovirus, enterovirus, coxsackievirus B, and poliovirus blends (LIGHT DIAGNOSTICS^™^, Millipore); respiratory virus-positives were further typed by monoclonal antibody for adenovirus, influenza A, influenza B, parainfluenza 1–3, or respiratory syncytial virus (LIGHT DIAGNOSTICS^™^, Millipore). PanEV-positive untypable specimens were examined by PCR sequencing, and immunostaining of infected cells with monoclonal antibodies specific for echovirus 4, 6, 9, 11, and 30, or coxsackievirus B1 to B6 for confirmation (S1 Fig). Negative results were determined by blind staining with PanEV and respiratory virus screen antibody.

### RNA & DNA extraction

To detect sewage viruses growing in the cell culture, viral RNA and DNA were extracted from cell culture lysates using a ZR viral RNA kit (ZYMO Research) and a QIAamp DNA mini kit (QIAGEN) according to the manufacturer’s instructions.

### Enterovirus molecular typing by CODEHOP PCR

PanEV-positive untypable specimens were analyzed by reverse transcription-seminested PCR (RT-snPCR), namely, the CODEHOP PCR method developed by Nix *et al*. [35] with modification. The primers used in this study are listed in S1 Table. Briefly, cDNA was synthesized in a 10-μl reaction mixture containing 5 μl of RNA, 10 mM deoxynucleoside triposphate (dNTP), 5x reaction buffer, 0.1 M dithiothreitol (DTT), 10 μM each of cDNA primer (primer AN32, AN33, AN34, and AN35), 40 U of RNasin, and 200 U of SuperScript III reverse transcriptase. Following incubation at 22°C for 10 min, 42°C for 60 min, and 95°C for 5 min, the total 10 μl RT mixture was then used in the first PCR reaction, consisting of primers 224 and 222, with 40 cycles of amplification (94°C for 30s, 42°C for 30s, 68°C for 90s). Then 1 μl of the first PCR product was added to a second PCR with primers AN89 and AN88. After 40 cycles of seminested amplification (94°C for 30s, 60°C for 30s, 68°C for 30s), the PCR product was separated by 1.5% agarose gel electrophoresis and the positive product (∼350 to 400 bp) was purified using a Gel/PCR DNA fragment extraction kit (Geneaid). The resulting DNA sequencing was performed with primer AN88 or AN89. The BigDye^®^ Terminator v3.1 Cycle Sequencing Kit (ABI, Foster City, USA) was used for sequencing with an ABI PRISM 3730XL Genetic Analyzer.

### Mammalian orthoreovirus PCR sequencing

Besides CODEHOP PCR sequencing, untypable PanEV-positive specimens were analyzed by PCR for identification of the conserved L1 region of MRV. The cDNA synthesis reaction mixture contained 5 μ1 of total extracted RNA, 10 pmol of each of the cDNA primers rv5F and rv6R (Table 1). Reactants included 4 μ1 of 5X reaction buffer (Invitrogen), 200 μM of each deoxynucleoside triphosphate (dNTP, Promega), 0.01 M dithiothreitol, 40 U of RNasin Ribonuclease Inhibitor (Promega), and 200 U of SuperScript III reverse transcriptase (Invitrogen) incubated at 94°C for 5 min, 20°C for 10 min, 42°C for 60 min, and 95°C for 5 min. Next, PCR was performed using 5 μ1 of RT reaction mixture, 5 μ1 of 10× buffer for KOD+ (TOYOBO), 0.2 mM of each dNTP, 1.5 mM of MgSO4, 10 pmol each of primers rv5F and rv6R, and 1 U of KOD+ DNA polymerase in a final volume of 50 μ1. The mixture was incubated at 94°C for 5 min followed by 40 cycles of 94°C for 30s, 52°C for 30s, and 68°C for 30s for amplification. The 416 bp PCR products separated by 1.5% agarose gel electrophoresis were purified using a Gel/PCR DNA fragment extraction kit (Geneaid). For MRV L1 region PCR-positive specimens, samples were subtyped by S1 region primer for MRV Types 1–3 (Table 1). Reverse transcription and PCR conditions were the same as the conditions for MRV L1 region, except the elongation time of PCR condition was extended to 90s. Slight variations in the size of the PCR products (~1169 to 1440 bp) were observed due to S1 gene length differences in the different subtypes. DNA sequencing was performed with the primer pairs of PCR of each region. The BigDye^®^ Terminator v3.1 Cycle Sequencing Kit (ABI, Foster City, USA) was used for sequencing with an ABI PRISM 3730XL Genetic Analyzer.

**Table 1:** Primers used for PCR amplification and sequence analysis.

Primer	Sequence 5' - 3'	Target	MRV specificity	Position
L1-rv5F^a^ L1-rv6R^a^	gCATCCATTgTAAATgACgAGTCTg CTTgAgATTAgCTCTAgCATCTTCTg	L1	All types	1930–1953 2249–2273
R1S1-F^b^ R1S1-R^b^	gCTATTCgCgCCTATggA ATACATgATCgTCCACggAg	S1	Type 1	1–18 1389–1408
R2S1-F^c^ R2S1-R^c^	gCTATTCgCACTCATgTC gATgAgTCgCCACTgTgCC	S1	Type 2	1–20 1422–1440
nR2S1-F^d^ nR2S1-R^d^	AATgggCCgTCAAgggAAAT AAATTgTACggCTgCgAACg	S1	Type 2	74–93 1224–1243
R3S1-F^e^ R3S1-R^e^	gCTATTggTCggATggAT gATgAAATgCCCCAgTgC	S1	Type 3	1–18 1399–1416

^a^Leary et al., (2002); primer position refers to the L1 sequence of T1L/53 (Accession: M24734)

^b^Primer position refers to the S1 sequence of TlL/53 (Accession:M35963)

^c^Primer position refers to the S1 sequence of T2J/55 (Accession:M35964)

^d^Primer position refers to the S1 sequence of BYD1 (Accession: DQ312301.1)

^e^Primer position refers to the S1 sequence of T3D/55 (Accession:M10262)

### Phylogenetic analysis

Sequence alignment was performed by ClustalW Multiple alignment using BioEdit software (version 7.0.5.3). Phylogenetic analysis was done using MEGA software (version 6.06), and phylogenetic trees were constructed by the neighbor-joining method based on MEGA software. The evolutionary distances were calculated using the Kimura 2-parameter method. Bootstrap value was computed on 1000 replicates and the significance of branch length was estimated by maximum likelihood.

### Nucleotide sequence accession numbers

The nucleotide sequences of the MRV obtained from environmental sewage described here have been deposited in the GenBank database under the following accession numbers: KR296769 to KR296785.

### Ethics statement

This study was reviewed and approved by the Centers for Disease Control, Department of Health, Taipei, Taiwan. Permission was obtained from the wastewater treatment plants before conducting the study.

## Results

### Environmental viral surveillance between 2012 and 2013

Between July 2012 and December 2013, a total of 300 sewage samples were collected from 10 representative wastewater treatment plants in Taiwan, which were chosen based on the population density of the relevant county (see map in Fig 1). The specimens were processed according to the WHO guidelines for environmental surveillance of poliovirus circulation, as described in Materials & Methods; S1 Fig shows the flow chart.

### Isolation and identification of viruses in sewage specimens

During the 18-month survey period, the sewage specimens were collected and analyzed. A total of 163 sewage specimens (54.3%) were positive for viral cultures and among them 268 isolates were identified (Table 2). After identification by immunofluorescent assay (IFA), 92 isolates (34.3%) showed enteroviruses but no poliovirus, and 82 isolates (30.6%) tested positive for adenoviruses (AdV). To type enteroviruses in the environment, enterovirus-positive specimens were assessed by IFA with monoclonal antibodies or CODEHOP PCR. The results showed that 84 isolates (91.3%) were identified as coxsackievirus B (CVB) and 8 isolates (8.7%) were echovirus (Echo). Among these, CVB4 was the most prevalent (27 isolates, 29.3%), followed by CVB3 (26 isolates; 28.3%), CVB2 (20 isolates, 21.7%), and CVB5 (11 isolates, 12.0%), whereas the proportions of echoviruses ranged between 1.1% to 3.3% of cases (Table 2).

**Table 2:** Regional distribution of viruses isolated from sewage samples in Taiwan (July 2012 to December 2013).

	No. of samples by region and city										
Virus isolation^a^	North (n = 90)			Central (n = 60)		South (n = 90)			East (n = 60)		
	Dihua	Neihu	Tamsui	Taichung	Douliu	Anping	Nanzi	Central District	Luodong	Hualien	Total
CVB 2	3	7	4		2		3			1	20
CVB 3	6	5	2	3	1		1		6	2	26
CVB 4	4	8	4	1	1		1		8		27
CVB 5	2	1	1		1	2			2	2	11
Echo 7		1			1						2
Echo 11						1	1		1		3
Echo 12	1			1							2
Echo 24									1		1
AdV	9	12	14	5	5	7	4	3	10	13	82
MRV	14	18	13	5	8	6	5	5	7	13	94
Total	39	52	38	15	19	16	15	8	35	31	268

^a^ CVB, coxsackievirus B; Echo, echovirus; AdV, Adenovirus; MRV, Mammalian orthoreovirus.

Interestingly, we found isolates from L20B cells were positive for PanEV antibody stain, but failed to be identified by CODEHOP PCR of enteroviruses. In order to ascertain whether they were enteroviruses, we performed PanEV RT-PCR which targets highly conserved sites in the 5'-nontranslated region (5'-NTR) of all members of enteroviruses [36] (The primers are listed in S1 Table). An unexpected PCR product was observed. After sequencing and blasting the NCBI database, the PCR products were identified as mammalian orthoreovirus (MRV). A possible reason for the unexpected identification of MRV by PanEV RT-PCR may be due to the similarity between the primer and L1 gene of MRV, as MRV was formerly classified as ECHO 10 [37]. In a study by Lelli *et al*., specific primers for the L1 region of all types of MRV were then applied to isolates which stained positive for the PanEV blend antibody in L20B cells [38]. Interestingly, 94 isolates (35.1%) were identified as MRV by MRV L1 region PCR (Table 2).

### Seasonal distribution of viruses isolated in sewage during monthly environmental surveillance in Taiwan

**Fig 2:**
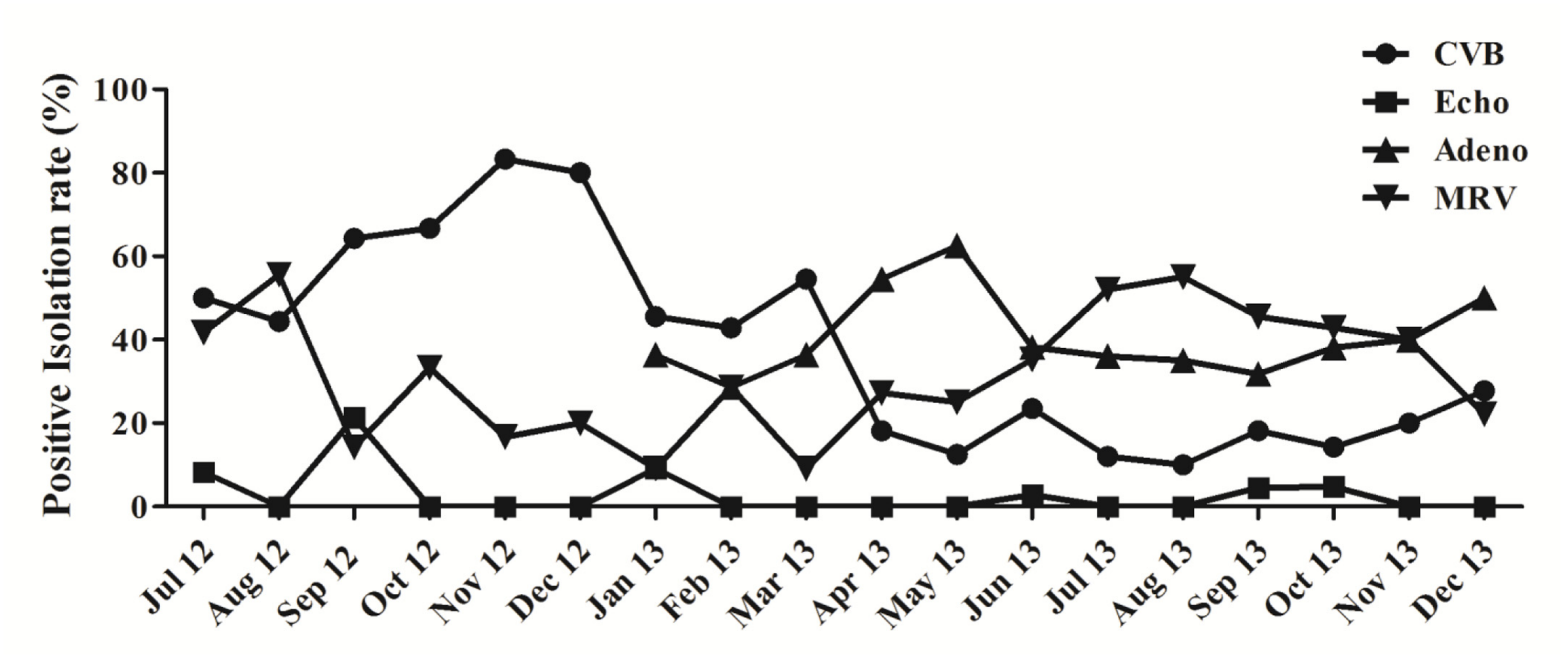
Monthly distribution of viruses isolated from routine environmental surveillance in Taiwan from July 2012 to December 2013. The positive isolation rates of the environmental virus are shown. CVB, coxsackievirus type B; Echo, echoviruses; AdV, adenovirus; MRV, mammalian orthreovirus. Adenoviruses were isolated when the A549 cell line was added in 2013.

**Fig 3:**
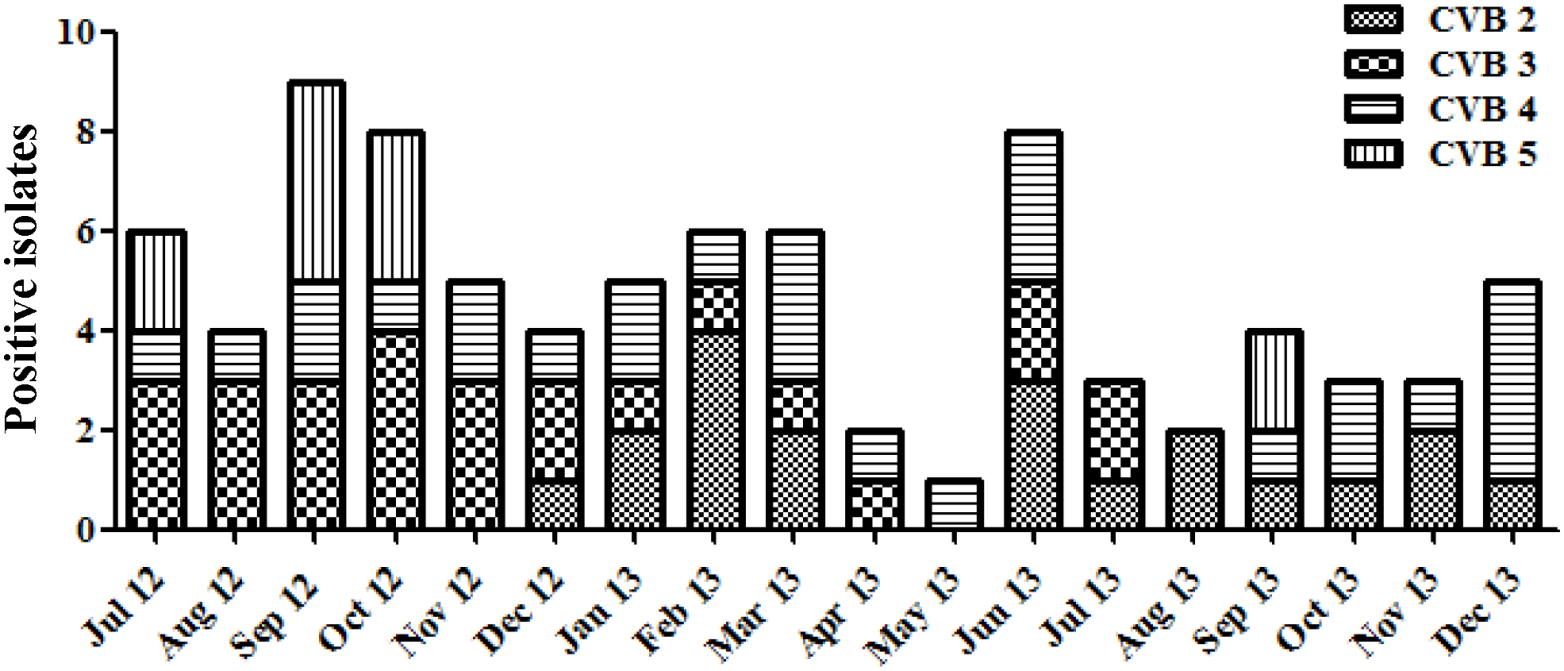
Monthly distribution of different subtypes of coxsackievirus B (CVB) isolated from environmental surveillance in Taiwan from July 2012 to December 2013. The positive isolates of the different subtypes of Coxsackievirus B are shown.

The monthly distribution of viruses isolated during the study period in Taiwan is shown in Fig 2. Except echoviruses, CVBs, AdVs, and MRVs were detected in all months during the study period. For CVBs, peaks were observed in the months from September to December in 2012, with lower activity in 2013. Serotypes CVB3 and CVB4 were more frequently detected, predominantly from July to December in 2012; however, serotypes CVB2 and CVB4 largely predominated in 2013 (Fig 3). The echoviruses were detected very rarely (8.7%) and were only sporadically isolated. For the AdVs, peaks were observed in April and May in 2013, whereas for the MRVs, a higher occurrence appeared between June and November in 2013.

### Phylogenetic analysis of mammalian orthoreoviruses from sewage specimens

**Fig 4:**
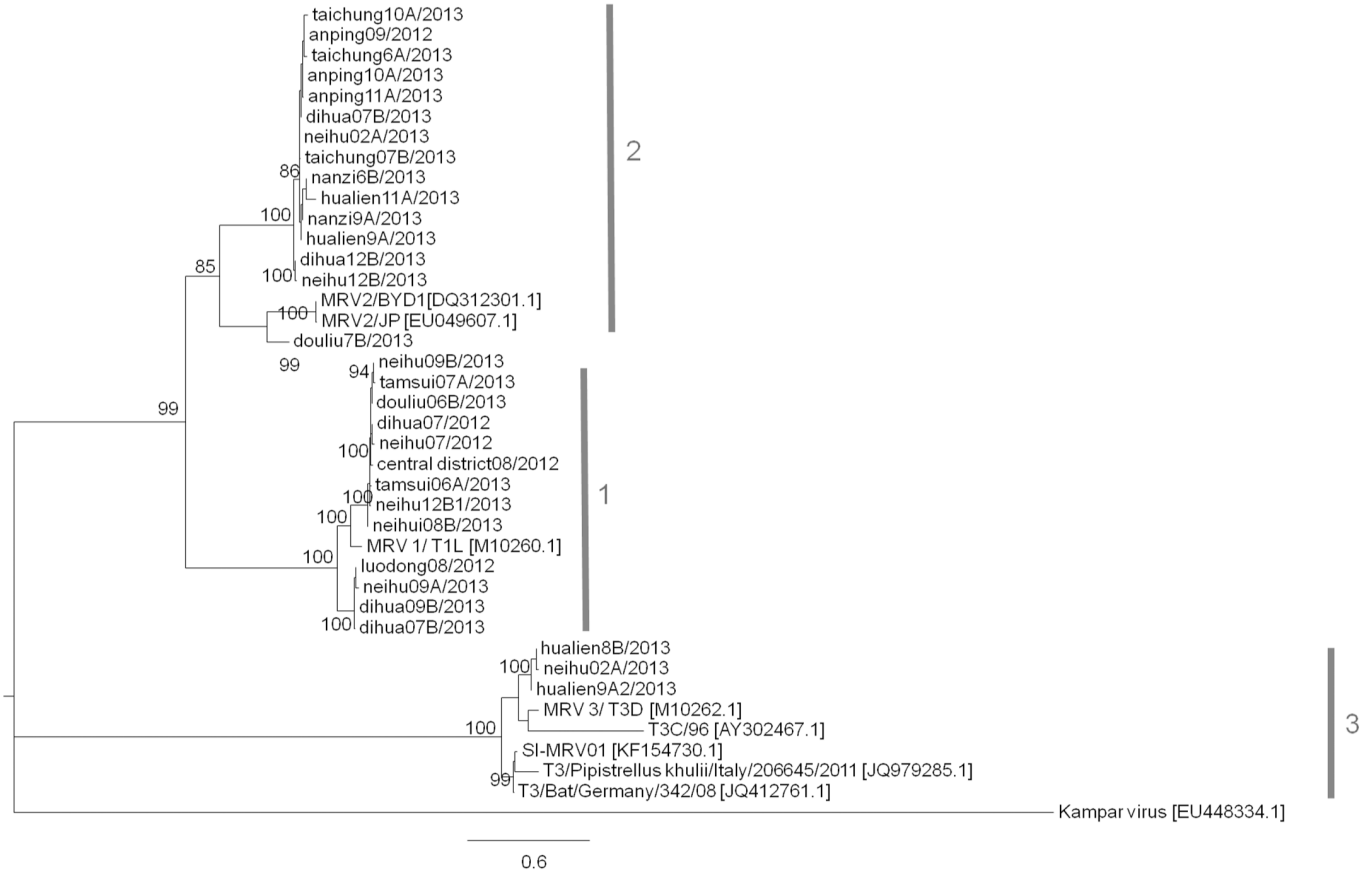
Phylogenetic analysis of the S1 gene of mammalian orthoreovirus. The S1 gene of MRV (nucleotides 147 to 1253) was analyzed. The unrooted tree was generated by the MEGA6 program using the neighbor-joining method with reference strains from the NCBI database. The GenBank accession number for the reference strains is given in brackets next to the virus name. The evolutionary distances were calculated using the Kimura 2-parameter method. Bootstrap value was computed on 1000 replicates and the significance of branch length was estimated by maximum likelihood. Only values higher than 70% are shown.

MRV are non-enveloped RNA viruses that contain 10 segmented double-stranded RNA genomes, including three large, three medium, and four small segments [39]. Previous studies have demonstrated a phylogenetic relationship between classical non-fusogenic MRV and the fusogenic reoviruses such as Nelson Bay reovirus (NBV) and baboon reovirus (BRV) based upon the S genome segments [40]. Therefore, to clarify the MRV genotypes isolated in this study, the S1 region (outer capsid gene) of the isolates was sequenced and compared with the sequences of MRV reference strains. Fig 4 shows the phylogenetic analysis of three serotypes of MRV based upon the S1 gene (nucleotides 147 to 1253) sequences. Thirty-one MRV were identified, including thirteen Type 1 (MRV1), fifteen Type 2 (MRV2), and three Type 3 (MRV3). Among these, MRV1 was more prevalent in 2012, whereas MRV1 and MRV2 co-circulated in 2013, and MRV3 was detected only in Neihu and Hualien, in northern and eastern Taiwan, in 2013. Sequence analysis and the phylogenetic tree showed that MRV1 and MRV2 persistently circulated in Taiwan and most MRV isolated in Taiwan was closely related to the reference strains isolated from patients with severe acute respiratory syndrome or meningitis reported previously in other countries.

## Discussion

In this study, viral surveillance of the sewage collected from 10 wastewater treatment plants throughout Taiwan was performed from July 2012 to December 2013. During this 18-month survey period, 300 raw sewage specimens were examined to detect the presence of viruses. The results showed that coxsackievirus type B, echoviruses, adenoviruses, and mammalian orthoreoviruses were isolated from the sewage specimens with a positive rate of 54.3%, but no poliovirus was found. Among these, enteroviruses (34.3%) and MRV (35.1%) predominated, followed by adenovirus (30.6%). Based on data provided by the WHO, at least 30% of concentrated sewage from grab samples can be expected to test positive for NPEV [41]. Therefore, the high rate of non-polio enteroviruses in the environment can be considered proof that the environmental samples were processed and analyzed appropriately to preserve virus infectivity. This study was undertaken to supplement poliovirus surveillance in Taiwan by monitoring the possible presence of wild-type (WPV) or vaccine-derived (cVDPV) poliovirus in wastewater with a view to obtaining further evidence supporting the maintenance of Taiwan's polio-free status.

The frequencies of detection of different enteroviruses differed by geographical area and year, but overall, coxsackievirus type B strains were isolated more often than echoviruses. These findings are similar to the results of some studies on sewage surveillance in other countries [42–45]. Among coxsackievirus B strains, CVB2, CVB3, and CVB4 were the most prevalent between July 2012 and 2013 in Taiwan; however, CVB1 and CVB6 were not isolated in this study. According to a surveillance report by Taiwan's Centers of Disease Control (Taiwan CDC), among the coxsackievirus B strains, CVB3 and CVB4 were more frequent in 2012, whereas CVB2 and CVB4 predominated in 2013. This phenomenon was in agreement with the results of this study that showed the majority subtype of coxsackievirus B in 2012 was CVB3, which shifted to CVB2 and CVB4 in 2013. The results indicated that the environmental virus strains reflect the viruses circulating in the population and highlight the potential risk of viruses spreading via wastewater. In addition, the rare isolation of coxsackievirus type A in our study might be related to the lower susceptibility of the cells used to isolate the viruses or the lower resistance of the viruses in the environment and the isolation process.

Besides the enteroviruses, adenoviruses and MRV were also identified by environmental surveillance in this study. Previous studies have reported adenoviruses and MRV in contaminated surface water and wastewater [46, 47]. Adenoviruses are non-enveloped, double strand DNA virus from the family *Adenoviridae* and are classified into species A to G with more than 57 identified genotypes. It has been shown that adenoviruses of species B (Ad 3, 7, 11& 14), species C (Ad 1, 2 & 5), and species E (Ad 4) are associated with acute respiratory disease [48–51]; adenoviruses of species F (Ad 40 & 41) are related to acute gastroenteritis in infants and children; species D (Ad 8, 19, 37, 54) is thought to cause epidemic keratoconjunctivitis; and species B (Ad 11, 21) has been linked to hemorrhagic cystitis [52–55]. According to data on clinical adenovirus isolation in Taiwan, Ad3, Ad7, and Ad4 were found mainly during outbreaks in southern Taiwan between 1999 and 2001, and Ad3 circulated in northern Taiwan between 2004 and 2005 [56, 57]. Recently, we reported that Ad3 was the dominant strain in southern Taiwan from 2002 to 2011, and a high incidence of co-infection with Ad2 was identified [58].

The unexpected identification of MRV by PanEV RT-PCR may be explained by the sequence similarity between the primers for enterovirus 5′-NTR and L1 gene of MRV, as MRV was formerly classified as ECHO 10. We found MRV was positive for PanEV antibody stain, but failed to be identified by CODEHOP PCR of enteroviruses. This may be attributed to the cross-reactivity of the PanEV Blend antibody toward reoviruses, as noted by the manufacturer in the instructions provided with the kit. According to our results, a high positive rate of MRV was found in our sewage specimens. MRV belongs to the Orthorovirus genus, *Spinareovirinae* subfamily, *Reovirus* family, and is also commonly termed reovirus. MRV are non-enveloped viruses that contain 10 segmented double-stranded RNA genomes, including three large (L1-3), three medium (M1-3), and four small (S1-4) segments [39]. It can be classified into three major serotypes: Type 1 Lang (T1L), Type 2 Jones (T2J), and Type 3 Dearing (T3D), which commonly cause asymptomatic infections or mild respiratory tract illness and enteritis in infants and children [37, 59, 60]. Other studies have reported a seropositive rate of more than 70% in 4-year-old children [27, 61]. Recently, a few novel MRV viruses were found in humans, such as, novel Type 2 MRV (MRV2TOU05), which seems to be closely related to porcine and human strains first isolated from 2 children with acute necrotizing encephalopathy in France; the mother of one patient also had influenza-like symptoms, and specific antibodies against MRV2TOU05 were detected [62]. Another novel Type 3 MRV was isolated from a child in the United States with meningitis. The virus also showed systemic spread and was found to produce lethal encephalitis in newborn mice after peroral inoculation [31]. Besides the novel MRV-infected pediatric cases, another novel MRV (Kampar virus) was identified from a throat swab of a 54-year-old patient with high fever, acute respiratory disease, and vomiting. Based on epidemiological tracing, there is a high probability that Kampar virus originated from fruit bats and is capable of causing human to human transmission according to the results of serological studies [63]. In previous studies, reoviruses were commonly found in environmental water sources, and human fecal contamination has been suggested as the source of the virus [64, 65]. Our PCR sequencing data of isolated MRV showed that mammalian orthoreoviruses Types 1, 2, and 3 were present in the environment. Sequence analysis also showed that MRV1 and MRV2 persistently circulated in Taiwan and most MRV isolated in Taiwan were closely related to the reference strains isolated from patients with severe acute respiratory syndrome or meningitis. These results suggest that the reoviruses isolated from sewage may have the potential to infect humans. However, no cases of MRV infection have been reported in Taiwan to date. Since the identification of MRV requires additional molecular analysis, it may be missed by routine viral identification. In addition, this study identified MRV from L20B cells, which are not normally used in routine settings. The etiologic agent remains unknown in many cases of encephalitis (32%-75%) [62]. MRV may be a potential risk factor with public health implications. Thus, L20B cell should be tested for routine viral isolation and PCR test is needed to identify MRV when MRV is suspected in human subjects.

In this report we provide an analysis of the environmental circulating viruses in Taiwan. Our results showed that Taiwan was poliovirus vaccine strain-free in the environment two years after the oral poliovirus vaccine was replaced by the inactivated poliovirus vaccine. Although our surveillance data were negative for poliovirus, long-term monitoring is still needed to allow prompt action should WPV ever be detected. In addition, we combined cell culture and RT-PCR to assay large volumes of sewage and identify the viruses, so that infectious viruses could be detected, thereby providing meaningful data that can be applied in public health risk assessments. This is the first study to report the prevalence of MRV in sewage in Taiwan. The observations made in this investigation highlight the potential risk of MRV infection in humans. This report also suggests that continuous periodic surveillance of environmental virus is necessary to prevent the outbreak of disease or reduce casualties. Finally, since MRV was frequently identified in our environmental specimens, it is imperative that human cases with suspected MPV infection be thoroughly evaluated.

## Supporting Information

S1 Fig The flow chart of the virus isolation and identification. (TIF)

S1 Table Primers used for cDNA synthesis, PCR amplification and sequence analysis of enteroviruses (DOCX)
