# Supplementary figures and images for: High Incidence of Mammalian Orthoreovirus Identified by Environmental Surveillance in Taiwan
Source: PLoS One. 2015 Nov 10;10(11):e0142745. doi: 10.1371/journal.pone.0142745 (PMC4640864; doi:10.1371/journal.pone.0142745)

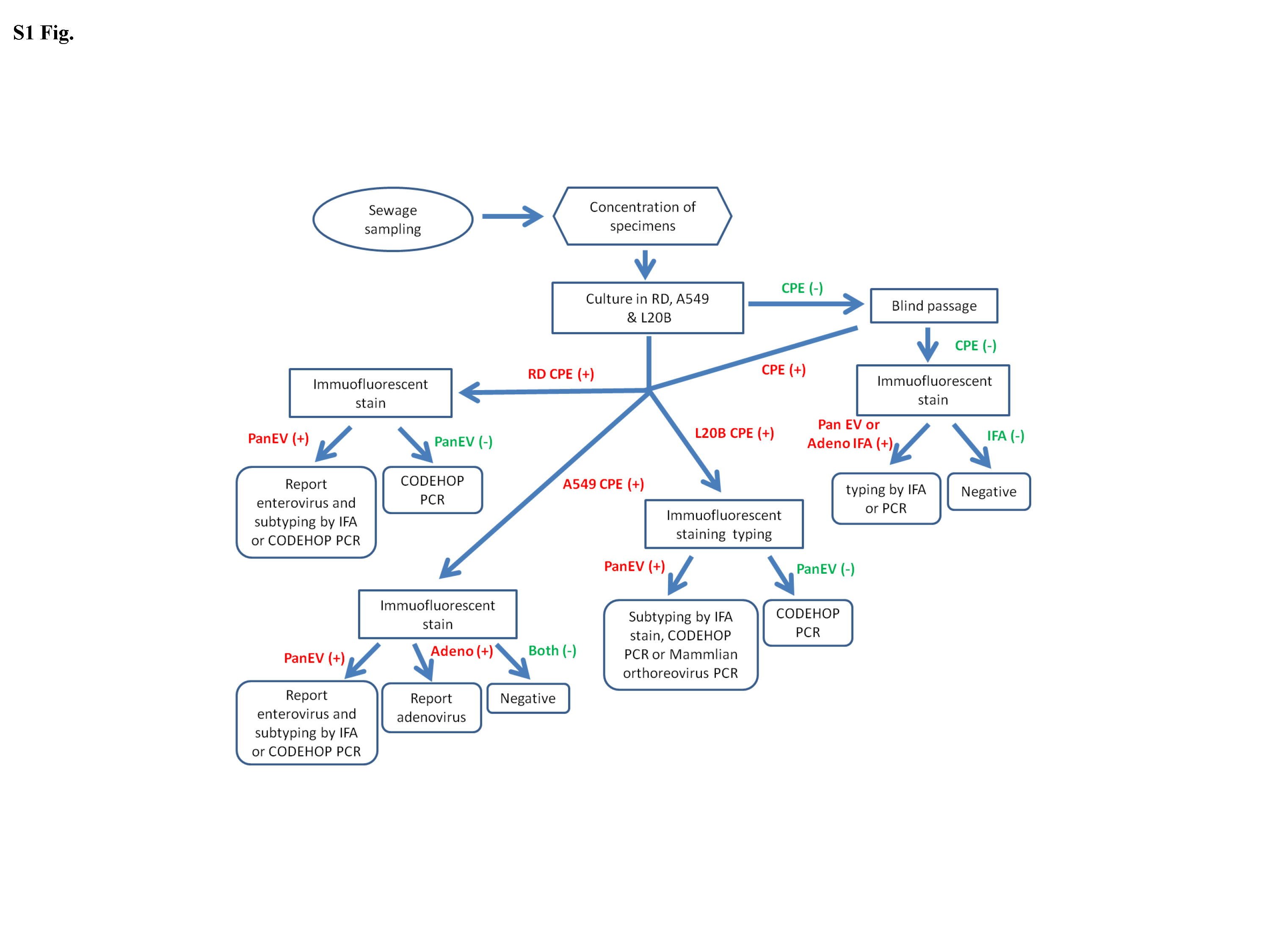

Supplement: S1 Fig — (TIF) [file pone.0142745.s001.tif]
