# Supplementary material for: High Incidence of Mammalian Orthoreovirus Identified by Environmental Surveillance in Taiwan
Source: PLoS One. 2015 Nov 10;10(11):e0142745. doi: 10.1371/journal.pone.0142745 (PMC4640864; doi:10.1371/journal.pone.0142745)
Supplement: S1 Table — (DOCX) [file pone.0142745.s002.docx]

**S1 Table. Primers used for cDNA synthesis, PCR amplification and sequence analysis of enteroviruses**

| **Primer** | **Sequence 5' - 3'** | **Target** | **Position** ^a^ |
| --- | --- | --- | --- |
| AN32  AN33 | gTYTgCCA  gAYTgCCA | VP1 | 3009–3002  3009–3002 |
| AN34  AN35 | CCRTCRTA  RCTYTgCCA |  | 3111–3104  3009–3002 |
| 224  222 | gCIATgYTIggIACICAYRT  CICCIggIggIAYRWACAT | VP3  VP1 | 1977–1996  2969–2951 |
| AN89  AN88 | CCAgCACTgACAgCAgYNgARAYNgg  TACTggACCACCTggNggNAYRWACAT | VP1 | 2602–2627  2977–2951 |
| EV-F  EV-R | CCCCTgAATgCggCTAATC  gATTgTCACCATAAgCAgC | 5′UTR | 450–468  580-596 |

^a^ The locations of all primers are those relative to the genome of PV1 Mahoney (GenBank accession number J02281).
